# Carbon Monoxide: A Context-Dependent Regulator of the Stress Axis

**DOI:** 10.3390/biom16060898

**Published:** 2026-06-18

**Authors:** Cesare Mancuso, Rosaria Santangelo

**Affiliations:** 1 Fondazione Policlinico Universitario Agostino Gemelli IRCCS, 00168 Rome, Italy; rosaria.santangelo@unicatt.it; 2 Department of Translational Medicine and Surgery, Section of Pharmacology, Università Cattolica del Sacro Cuore, Largo F. Vito, 1, 00168 Rome, Italy; 3 Department of Basic Biotechnological Sciences, Intensivological and Perioperative Clinics, Università Cattolica del Sacro Cuore, Largo F. Vito, 1, 00168 Rome, Italy

**Keywords:** ACTH, adrenal, bilirubin, biliverdin, biliverdin reductase, CRH, glucocorticoids, heme oxygenase, hypothalamus, pituitary

## Abstract

Carbon monoxide (CO) is a gasotransmitter generated by heme oxygenase (HO) isoforms during heme catabolism. The inducible HO-1 produces CO under conditions of redox imbalance, such as oxidative stress and inflammation. On the other hand, HO-2 constitutively generates CO, primarily during the physiological turnover of heme. Extensive evidence indicates that CO exerts autocrine effects by targeting hemoproteins, including soluble guanylyl cyclase, cyclooxygenase, and cytochromes. Furthermore, CO regulates many biological processes within the brain, including mitochondrial biogenesis, potassium channel activity, mitogen-activated protein kinase and phosphatidylinositol-3-kinase/Akt signaling. It also controls the activity of transcription factors, such as hypoxia-inducible factor-1 and peroxisome proliferator-activated receptor-γ. Through these mechanisms, CO modulates inflammatory gene expression, promotes anti-apoptotic signaling, and contributes to local stress responses. Conversely, CO produced in the hypothalamus inhibits the stress-induced release of corticotropin-releasing hormone and arginine vasopressin under pro-inflammatory conditions, resulting in reduced adrenocorticotropin hormone release and cortisol secretion from the anterior pituitary and adrenal cortex, respectively. Moreover, hypothalamic CO acts in a paracrine manner to modulate glucocorticoid release during psychological stress, including restraint or water deprivation. Together, these findings support the view that endogenous CO is a key modulator of the stress axis, exerting pleiotropic effects that integrate neuroendocrine, immune, and metabolic responses.

## 1. Introduction

The term “stress” was initially employed within the domain of physics, where it was defined as the force exerted on a body resulting in its deformation. In this context, stress is measured in units of force per area, for example, in Newtons per square inch [1]. Subsequently, the term was adopted in the biological sciences, while retaining its original meaning, namely an event capable of disrupting the homeostasis of an organism [1]. At the organ-system level, stress is broadly categorized into acute and chronic forms, based on duration and physiological impact. Acute stress elicits a rapid and adaptive activation of both the hypothalamic–pituitary–adrenal (HPA) axis and the sympatho-adrenal-medullary (SAM) system. Within the HPA axis, this response involves the sequential release of corticotropin-releasing hormone (CRH), adrenocorticotropic hormone (ACTH) and cortisol from hypothalamus, anterior pituitary and adrenal cortex, respectively [2]. Simultaneously, catecholamines, such as epinephrine and norepinephrine, are secreted by the adrenal medulla in the bloodstream [3,4]. This neuroendocrine cascade enhances cardiovascular output, glucose availability, and arousal, thereby facilitating immediate survival responses (see also Section 2.3) [5]. Acute stressors include car accidents, possible job losses, or psychosocial threats [6,7]. In contrast, chronic stress involves a prolonged or repeated activation of the HPA axis. This may result in dysregulation of glucocorticoid (GC) secretion, attenuated negative feedback sensitivity, and structural and functional changes in key brain regions, such as the hippocampus, prefrontal cortex, and amygdala [8,9]. Chronic HPA axis activation has been associated with flattened diurnal cortisol rhythms, neuroinflammation, and increased susceptibility to affective disorders, metabolic dysfunctions, and cognitive decline [10,11]. At the cellular level, the terms “oxidative stress” and “nitrosative stress” are employed when the homeostatic disturbance is due to reactive oxygen or nitrogen species (ROS or RNS, respectively). This overload of free radical species commonly results in extensive damage to DNA, lipids, or proteins. When persistent or irreversible, these modifications impair cellular function and may ultimately lead to cell death [12,13,14].

**Figure 1:**
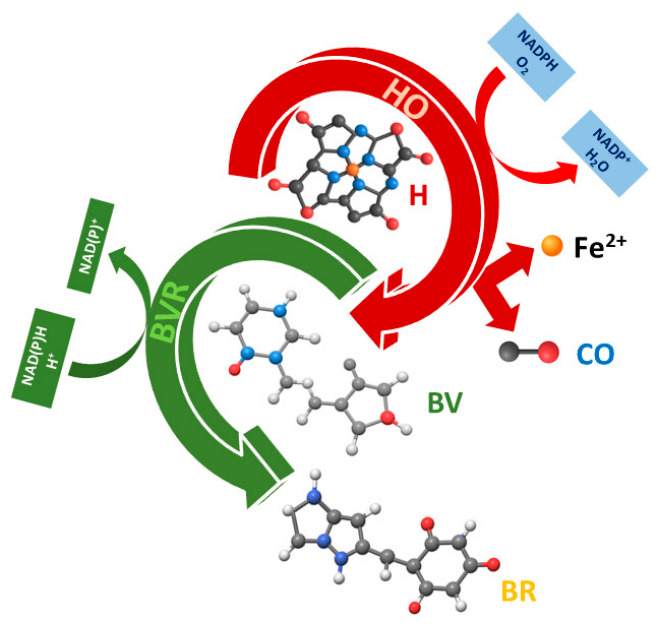
Heme oxygenase (HO) catalyzes the oxidation of the α-meso-carbon bridge of ferroprotoporphyrin-IXα (heme, H) leading to the formation of ferrous iron (Fe^2+^), carbon monoxide (CO), and biliverdin-IXα (BV) in equimolar amounts (red wheel). Heme oxygenase functions along with biliverdin reductase-A (BVR), which, by reducing BV to bilirubin-IXα (BR), catalyzes the final step of heme metabolism in mammals (green wheel). For further details, see Section 3.

Heme oxygenase (HO) is a key enzyme in heme metabolism, catalyzing the conversion of ferroprotoporphyrin-IXα into ferrous iron (Fe^2+^), carbon monoxide (CO), and biliverdin-IXα (BV) [15] (Figure 1). Two HO isoforms have been described, and named HO-1 and HO-2. Among these isoforms, the inducible HO-1 is upregulated under conditions of redox imbalance caused by excessive production of free radicals and pro-inflammatory mediators, thereby playing a pivotal role in the cellular stress response. Conversely, the constitutive HO-2 is mainly involved in the physiologic turnover of heme and gas sensing [15,16]. Heme oxygenase functions along with biliverdin reductase-A (BVR), which catalyzes the final step of heme metabolism in mammals by reducing BV to bilirubin-IXα (BR) [17,18,19] (Figure 1). The enzymatic products of HO/BVR activities, CO and BV/BR, act as endogenous signaling molecules with effective antioxidant and cytoprotective properties across multiple cell types and tissues [20,21,22,23,24]. Scientists had underestimated the potential role of endogenous CO until 1993, when Verma et al. proposed CO as a novel neuromodulator involved in long-term potentiation and synaptic plasticity (see also Section 4.2) [25]. Following this important observation, subsequent studies provided evidence for the key role of endogenous CO in regulating brain functions, including glutamate release and signaling, memory, nociception and neuropeptide secretion [25,26,27,28,29,30,31]. Carbon monoxide molecular targets include heme-containing proteins and redox-sensitive pathways, which regulate neuronal excitability, vascular tone, and cytoprotective mechanisms [32,33,34,35,36]. In addition to the nervous system, CO has been shown to modulate non-adrenergic, non-cholinergic gastrointestinal relaxation, immune responses, and renal function [16,24,37].

In this context, the present review aims to clarify the physiological and molecular mechanisms through which CO regulates the release of hypothalamic and pituitary neuropeptides involved in the stress response, and to discuss the consequent adaptations occurring in downstream target glands and tissues.

## 2. The Stress Axis

### 2.1. The Hypothalamic-Pituitary Unit

**Figure 2:**
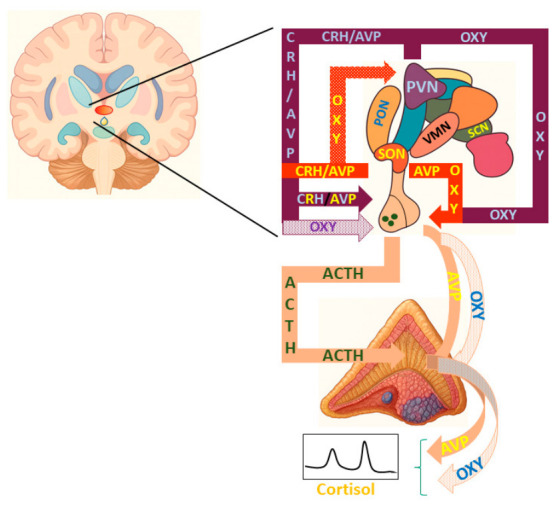
The hypothalamus integrates autonomic, metabolic, and endocrine functions and contains nuclei essential for stress regulation, particularly the supraoptic nucleus (SON) and the paraventricular nucleus (PVN). Neurons in the PVN and SON synthesize corticotropin-releasing hormone (CRH) and arginine vasopressin (AVP), the primary hypothalamic signals driving stress axis activation. Parvocellular PVN neurons release CRH into the median eminence, from which it reaches the anterior pituitary via the portal circulation to stimulate adrenocorticotropic hormone (ACTH) secretion. Arginine vasopressin from magnocellular PVN and SON neurons further enhances ACTH release and is also secreted into the systemic circulation through the posterior pituitary. Under basal conditions, CRH and AVP are secreted in circadian pulses that regulate ACTH-driven cortisol production. Rising glucocorticoid levels provide negative feedback on the hypothalamus and pituitary to prevent excessive HPA activation. Both SON and PVN also contain oxytocinergic neurons, which modulate the stress response through multiple pathways: (i) activation of GABAergic interneurons that inhibit CRH release, (ii) oxytocin (OXY)-mediated reduction of ACTH secretion via actions at the median eminence and anterior pituitary, and (iii) direct suppression of cortisol synthesis in the adrenal cortex. Together, these pathways illustrate how hypothalamic circuits coordinate neuroendocrine mechanisms to regulate stress axis activity. Representative hypothalamic nuclei are shown (PON, preoptic nucleus; SCN, suprachiasmatic nucleus; VMN, ventromedial nucleus). The colors of the arrows correspond to those of the hypothalamic nuclei, pituitary gland, and adrenal gland. The dots in the adenohypophysis represent the corticotroph cells.

At the neuroendocrine level, the hypothalamus coordinates autonomic and endocrine responses to systemic challenges. Within the hypothalamus, the paraventricular and supraoptic nuclei (PVN and SON, respectively) represent the principal node of the HPA axis, integrating neural and humoral stress-related signals and initiating downstream pituitary activation [2,38,39]. This hypothalamic-pituitary communication is mediated primarily through the median eminence and the hypophyseal portal circulation, which allow hypothalamic neuropeptides to reach the anterior pituitary (adenohypohysis) in a highly regulated manner [39,40]. In response to stress, parvocellular neurons of the PVN synthesize and release CRH into the median eminence. Arginine vasopressin (AVP), produced predominantly by magnocellular neurons of the PVN and SON, can also reach the adenohypophysis, through the hypophyseal portal circulation, and potentiates CRH-induced ACTH secretion from corticotroph cells [39,41,42] (Figure 2). In parallel, magnocellular axons project to the posterior pituitary (neurohypophysis), where AVP is released into the systemic circulation, thus contributing to fluid balance and broader homeostatic adaptation during stress [39,42] (Figure 2).

Under basal conditions, HPA axis activity follows circadian and ultradian rhythms, with higher-amplitude CRH/AVP-ACTH pulses in the morning in humans. Stress amplifies and synchronizes hypothalamic secretory activity, increasing ACTH release and, in turn, cortisol secretion from the adrenal cortex [43]. Cortisol supports stress adaptation by mobilizing energy substrates through gluconeogenesis, lipolysis, and proteolysis, while also modulating inflammatory responses [3,44]. Rising circulating GC levels then engage negative feedback mechanisms at hypothalamic, pituitary, and extra-hypothalamic levels, restraining further CRH and ACTH release and preventing prolonged GC exposure, which may contribute to mood disturbances and neurodegenerative processes [41,42] (see also Section 2.3).

Oxytocinergic signaling provides an additional modulatory brake on HPA-axis activation. Oxytocin-producing neurons in the PVN and SON can influence stress axis activity through both central and peripheral mechanisms. These include facilitation of GABAergic inhibition of parvocellular CRH neurons, modulation of corticotroph cell responsiveness at the anterior pituitary, systemic release from the posterior pituitary, and possible direct actions at the adrenal cortex that may suppress cortisol synthesis [45,46,47,48] (Figure 2). Thus, oxytocin helps integrate neuroendocrine stress responses with inhibitory circuits that limit excessive HPA axis activation.

### 2.2. Extra-Hypothalamic Brain Areas

**Figure 3:**
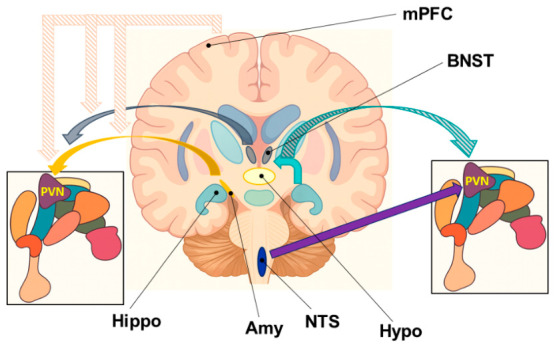
Schematic representation of the major extra-hypothalamic regions that modulate hypothalamic–pituitary–adrenal (HPA) axis activity. The amygdala (Amy) provides excitatory drive to the paraventricular nucleus (PVN), enhancing CRH release in response to emotionally salient stimuli. In contrast, the hippocampus (Hippo) mediates negative feedback via glucocorticoid-sensitive circuits and inhibits the HPA axis through intermediary GABAergic relays. A key component of this inhibitory pathway is the anteroventral bed nucleus of the striae terminalis (BNST), which receives hippocampal-dependent input through the septum and subiculum and contains dense GABAergic populations that suppress PVN CRH neurons. The medial prefrontal cortex (mPFC) negatively modulates stress responses either directly or through descending control of Amy and BNST output. In addition, the nucleus of the solitary tract (NTS) supplies viscerosensory catecholaminergic inputs that shape autonomic and neuroendocrine contributions to PVN regulation. The colors of the arrows correspond to those of the brain areas described. Full arrows, stimulation; dashed arrows, inhibition.

Extra-hypothalamic regulation of the HPA axis is mediated by a distributed corticolimbic–brainstem network that includes the amygdala, hippocampus, bed nucleus of the stria terminalis (BNST), medial prefrontal cortex (mPFC), and nucleus of the solitary tract (NTS) (Figure 3). The amygdala, particularly its central and basolateral nuclei, generally facilitates HPA axis activation through polysynaptic inputs to the PVN. By responding strongly to emotionally salient or threat-related stimuli, this brain area can promote CRH release and contribute to sustained HPA activation during chronic stress or affective disorders [2]. In contrast, the hippocampus, especially the ventral and CA1 regions, participates in GC-sensitive negative feedback. Because principal hippocampal outputs are glutamatergic, their inhibitory influence on PVN CRH neurons is thought to rely on interposed GABAergic relays, including dense inhibitory populations within the ventral BNST [49,50,51]. Accordingly, damage or dysfunction in the hippocampus, as may occur during chronic stress or neurodegeneration, can weaken feedback inhibition and prolong HPA axis activation [49,50].

Therefore, the BNST acts as an integrative interface between limbic inputs and hypothalamic stress-effector systems. Depending on its neurochemical organization, the BNST can modulate PVN CRH neurons and is particularly relevant to sustained stress responses [51,52]. The mPFC provides additional top-down modulation of HPA axis activity. While prelimbic regions generally exert inhibitory control through descending regulation of amygdala and BNST circuits, the contribution of the infralimbic cortex appears more context-dependent and may vary according to stressor type and duration [52,53,54]. Finally, brainstem nuclei, particularly the NTS, convey visceral and immune-inflammatory signals to the hypothalamus. For example, blood-borne interleukin-1 (IL-1) can activate parvocellular PVN neurons through ascending catecholaminergic projections from the NTS, including noradrenergic A2 inputs, thereby promoting ACTH/GC release during systemic stress [55,56,57] (Figure 3). Collectively, these regions form a distributed regulatory network that shapes the magnitude, duration, and termination of HPA axis activation in response to internal and external challenges. Dysregulation of this circuitry may contribute to stress-related disorders, including anxiety, depression, and post-traumatic stress disorder.

### 2.3. The Adrenal

The adrenal glands are a major peripheral effector of the stress system, integrating HPA axis output with SAM activation. Within the adrenal cortex, ACTH acts primarily on zona fasciculata cells through melanocortin 2 receptor (MC2R) signaling, engaging cyclicAMP/protein kinase-A (cAMP/PKA)-dependent steroidogenic pathways that culminate in cortisol synthesis in humans [58,59,60,61]. Cortisol promotes adaptation to stress by mobilizing energy substrates and supporting the restoration of homeostasis. Moreover, cortisol also restraining hypothalamic and pituitary drive through negative feedback mechanisms [3,59,62]. In parallel, the adrenal medulla functions as the endocrine arm of the sympathetic nervous system. Preganglionic sympathetic input activates chromaffin cells through nicotinic acetylcholine receptors, leading to the rapid systemic release of epinephrine and norepinephrine [63,64,65]. These catecholamines increase cardiovascular output, bronchodilation, and glucose availability, thereby complementing the slower and more sustained actions of GCs [63,66].

Thus, coordinated adrenal cortical and medullary outputs allow the organism to mount a time-sensitive response to physical and psychological challenges. Conversely, impaired adrenal function can compromise stress adaptation and systemic homeostasis [67].

## 3. Endogenous Carbon Monoxide

### 3.1. Carbon Monoxide Synthesis

**Figure 4:**
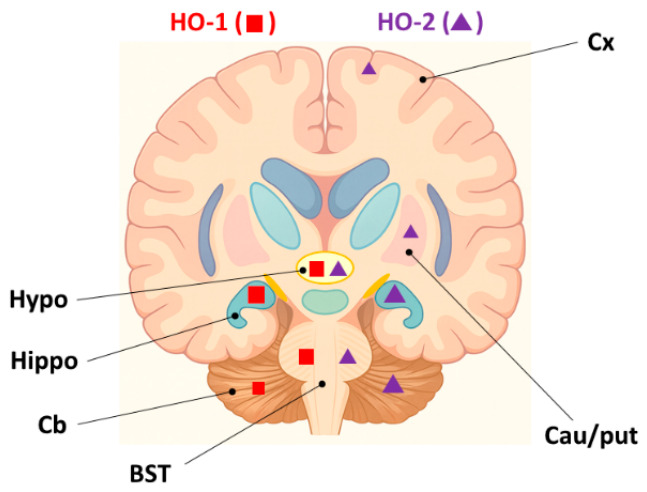
Distribution of heme oxygenase isoforms in the brain. Heme oxygenase-1 (HO-1, red squares) shows low, region-specific expression, mainly in the dentate gyrus of hippocampus (Hippo) and in hypothalamic ventromedial and paraventricular nuclei (Hypo). Heme oxygenase-1 is inducible in glial cells under oxidative stress. In contrast, heme oxygenase-2 (HO-2, blue triangles) is the predominant neuronal isoform, broadly expressed in the olfactory bulb, cortex (Cx), hippocampus (pyramidal neurons of CA1–CA3), hypothalamus (ventromedial and supraoptic nuclei), caudate/putamen (Cau/put), cerebellum (Cb), and brainstem (BST). Marker size indicates associated expression levels of each isoform.

Carbon monoxide, a diatomic gaseous molecule, is produced through the HO-mediated oxidative cleavage of heme groups from hemoproteins. This process occurs in four sequential, energy-dependent steps. In the presence of molecular oxygen and reducing equivalents supplied by NADPH-cytochrome P450 reductase, HO catalyzes the oxidation of the α-meso-carbon bridge of heme, resulting in the generation of equimolar amounts of Fe^2+^, BV and CO [15,16] (Figure 1). Since HO isoforms are widely distributed across tissues, CO can be regarded as a ubiquitous signaling molecule. In the liver and spleen, organs primarily responsible for the degradation of senescent erythrocytes and the recycling of hemoglobin, CO production mainly depends on HO-1 activity [15]. In contrast, CO generated within the brain originates predominantly from HO-2, the isoform most abundantly expressed in neurons, although low levels of HO-1 expression have also been detected in specific neuronal populations [15]. In the rat CNS, HO-1 expression is generally low and confined to discrete neuronal clusters, including those in the dentate gyrus of the hippocampus and in the ventromedial and paraventricular nuclei of the hypothalamus [15] (Figure 4). Heme oxygenase-1 is also found in glial cells, where oxidative stress strongly induces its expression [68]. Heme oxygenase-2, on the other hand, represents the predominant isoform in the brain, but is also expressed in peripheral tissues, such as testes, kidneys and blood vessels [15]. In the rat brain, HO-2 is present in several neuronal populations, including those located in the olfactory bulb, cortex (pyramidal neurons), hippocampus (pyramidal neurons of CA1–CA3), hypothalamus (ventromedial and supraoptic nuclei), basal ganglia (caudate-putamen), cerebellum, and brainstem [15,69] (Figure 4). Lastly, both HO-1 and HO-2 have been identified in adrenal zona fasciculata cells, suggesting a potential role for CO in the autocrine modulation of adrenal function [70].

**Figure 5:**
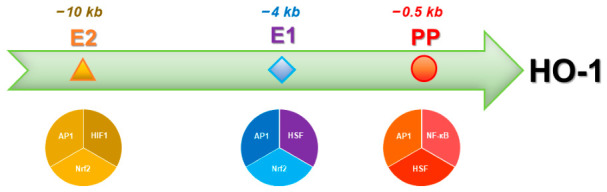
Upstream regulatory architecture of the HO-1 gene. Three major enhancer clusters, named PP, E1, and E2 are located within the 5′-flanking region of the HO-1 gene. These elements function as binding sites for multiple transcription factors, including heat shock factor (HSF), activator protein-1 (AP-1), nuclear factor-κB (NF-κB), nuclear factor erythroid 2-related factor 2 (Nrf2), and hypoxia-inducible factor-1 (HIF-1). Both AP-1 and NF-κB primarily mediate HO-1 induction in response to immune-inflammatory signals, whereas Nrf2 serves as the principal activator under oxidative or electrophilic stress, driving robust transcriptional upregulation of HO-1.

Intracellular CO levels depend on the extent of both HO-1 and HO-2 modulation. The expression of HO-1 is strictly controlled at transcriptional, epigenetic, and post-transcriptional levels, allowing cells to adapt accurately to oxidative, inflammatory, and metabolic stress [71,72,73]. Three upstream enhancer clusters, named PP, E1, and E2, located ~0.5 kb, ~4 kb, and ~10 kb upstream of the HO-1 transcription start site, respectively, have been identified within its 5′-flanking region [72,73] (Figure 5). These regulatory elements serve as binding sites for multiple transcription factors, including heat shock factor (HSF), activator protein-1 (AP-1), nuclear factor-κB (NF-κB), nuclear factor erythroid 2-related factor 2 (Nrf2), and hypoxia-inducible factor-1 (HIF-1) [72,73] (Figure 5). Among these, AP-1 and NF-κB predominantly promote HO-1 induction in response to immune-inflammatory stimuli [73,74,75]. On the other hand, Nrf2 plays a central role in up-regulating HO-1 during oxidative or electrophilic stress [73,74,75]. In light of this, pro-inflammatory stimuli, such as bacterial lipopolysaccharide (LPS) and IL-1, trigger phosphorylation and degradation of IκB proteins, allowing NF-κB (typically p65/p50 heterodimers) to translocate to the nucleus and bind κB motifs in target promoters [72,73]. In parallel, LPS and IL-1 activate mitogen-activated protein kinase (MAPK) pathways, including JNK and p38, which phosphorylate AP-1 components, such as c-Jun and c-Fos, enhancing their transcriptional activity [75,76]. Once activated, NF-κB and AP-1 interact with the PP and E1/E2 enhancer clusters of the HO-1 promoter, cooperating with additional transcription factors, such as Nrf2 and Sp1, to drive transcription [77]. Conversely, Nrf2, a redox-sensitive transcription factor, is primarily responsible for HO-1 induction in the event of cellular redox imbalance, such as increased ROS production or exposure to reactive chemicals [72,73].

**Figure 6:**
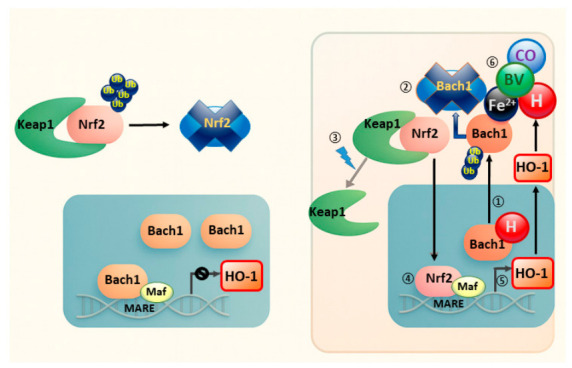
Regulation of HO-1 transcription by Bach1 under basal conditions and by Nrf2 during heme excess or electrophilic/oxidative stress. (**Left panel**): Under non-stress conditions, Bach1 heterodimerizes with small Maf proteins and binds Maf recognition elements (MARE) within the E1/E2 enhancer regions of the HO-1 gene. Bach1 recruits co-repressor complexes and histone-modifying enzymes that maintain chromatin in a closed configuration, thereby suppressing HO-1 transcription. (**Right panel**): Elevated intracellular heme binds specific heme regulatory motifs in Bach1 ① inhibiting its DNA-binding activity, promoting its dissociation from the enhancer, and triggering nuclear export and proteasomal degradation ②. Oxidative or electrophilic stress disables Keap1-mediated degradation of nuclear factor erythroid 2-related factor 2 (Nrf2) ③ allowing it to accumulate and translocate into the nucleus. Finally, Nrf2 then heterodimerizes with small Maf proteins, occupies the same MARE sites freed by Bach1, and recruits co-activators that open chromatin structure ④. This replacement of Bach1 by Nrf2 drives robust HO-1 transcription ⑤ leading to HO-1 upregulation and formation of carbon monoxide (CO), biliverdin (BV) and ferrous iron (Fe^2+^) ⑥. Reproduced, with modifications, from Song et al. *Front. Physiol.* 2023 [80].

Under basal conditions, Nrf2 is sequestered in the cytoplasm by Keap1, which promotes its ubiquitination and proteasomal degradation. Oxidative or electrophilic modification of critical Keap1 cysteines disrupts this complex, stabilizing Nrf2 and enables its nuclear translocation [72,73]. In the nucleus, Nrf2 heterodimerizes with small Maf proteins and binds Maf recognition elements (MARE) located within the E1/E2 clusters to promote HO-1 transcription [72,73] (Figure 6). To prevent inappropriate HO-1 induction under basal conditions, the promoter is actively repressed by Bach1, a transcriptional inhibitor that competes with Nrf2 for heterodimerizing with Maf and binding to MARE [78,79,80]. Under pro-oxidant conditions, elevated intracellular heme binds to Bach1, promoting its nuclear export and degradation, thereby relieving repression and coupling heme accumulation to HO-1 induction [78,79,80] (Figure 6). Mitogen-activated protein kinase signaling pathways (ERK, JNK, p38) further modulate Nrf2 activity, fine-tuning the transcriptional output [72,73].

Besides transcriptional control, epigenetic mechanisms, including DNA methylation and histone acetylation, shape chromatin accessibility and influence HO-1 basal promoter competence [81]. Post-transcriptionally, several microRNAs (e.g., miR-217, miR-155) bind the HO-1 mRNA, modulating its stability and translation in cell-specific contexts [82,83]. Moreover, post-translational processing also contributes to functional diversity. Heme oxygenase-1 is synthesized as a ~32 kDa endoplasmic reticulum–anchored protein, although stress conditions can trigger proteolytic cleavage. This last modification generates truncated HO-1 forms able to translocate to the mitochondria or nucleus, where they exert non-canonical cytoprotective and signaling functions [72,84].

Heme oxygenase-2 is the constitutive isoform and is mainly involved in the physiologic turnover of heme [15,85]. In contrast to HO-1, the HO-2 promoter lacks antioxidant responsive elements (AREs), making it largely unresponsive to oxidative or electrophilic stress [15]. However, studies by Mahin Maines’ group showed that, in the promoter region, the rat HO-2 gene contains a consensus sequence of the GC-responsive element (GRE, between nucleotides −9723 to −9716), that makes it sensitive to adrenal GCs [15,86,87]. However, this GRE is not a strong transcriptional promoter; therefore, HO-2 overexpression may also depend on both an increased translation rate and protein stabilization [86,88,89]. Finally, HO-2 is highly expressed in oxygen-sensing tissues, including the carotid body and brainstem, where it acts as an intrinsic oxygen sensor via heme-regulatory motifs (HRMs) within the protein (see below) [15,71,90].

### 3.2. Carbon Monoxide Delivery Systems

Several experimental approaches have been developed to investigate the biological and pharmacological actions of CO. Heme administration represents an indirect strategy, as it stimulates endogenous CO production by inducing HO-1 and increasing substrate availability for both HO-1 and HO-2. Nevertheless, its interpretation is complicated by the concomitant modulation of Fe^2+^ release, BV formation, and other heme-dependent processes [15,16,20,21]. This experimental approach was widely used in early studies, in which pharmacological inhibition of HO activity was required to determine whether the effects of heme/hemin supplementation were truly dependent on HO activation. Furthermore, pharmacological HO inhibitors have also been used to evaluate the biological effects of basal HO-derived CO production [26,27,28,29,30,31,36].

This indirect approach underwent a major shift in the early 2000s. This was due to the development and biological application of carbon monoxide-releasing molecules (CORMs) able to release CO and reproduce CO-like biochemical effects [32,34]. These CO donors provided a pharmacological tool for delivering CO in a more controlled manner. The earliest compounds were transition metal-based CORMs, mostly metal carbonyl complexes such as ruthenium-, manganese- and iron-containing compounds. More recent and promising agents are metal-free or organic CORMs; photoCORMs, which release CO upon light activation; enzyme-triggered CORMs, activated by specific metabolic or enzymatic pathways; and CO-releasing materials or nanomaterials, designed to improve solubility, targeting, and release kinetics [32,34]. The main advantages of CORMs are their tunable CO-release profiles and the potential for spatially and temporally controlled delivery. The major drawbacks include variable stability, possible toxicity of metal or organic by-products, and the risk that biological effects may derive from the carrier molecule rather than from CO itself [32,34].

Finally, direct administration of gaseous CO to cells or tissues allows a more straightforward assessment of CO-dependent responses, but it requires careful control of concentration, exposure time, diffusion, and safety conditions. In particular, the amount of gaseous CO that dissolves in cell culture media may vary depending on the chemical composition of the medium, gas–liquid exchange, and the partial pressure of CO. This issue may prevent an accurate and unambiguous quantification of CO concentration in the culture system, unless gas exposure conditions are carefully standardized [32,34]. Taken together, these methods should be viewed as complementary rather than interchangeable tools for dissecting CO biology.

### 3.3. Carbon Monoxide Targets

**Figure 7:**
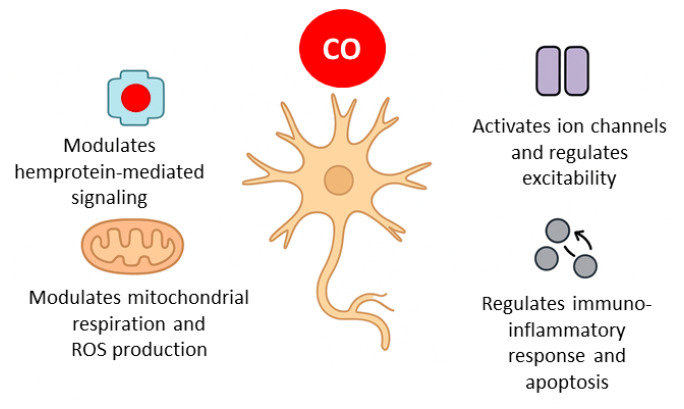
Molecular targets and signaling pathways underlying the neuromodulatory actions of endogenous carbon monoxide (CO). Early studies identified soluble guanylyl cyclase (sGC) as a primary CO target in the nervous system. However, subsequent evidence demonstrated that CO is only a weak activator of sGC compared with nitric oxide, suggesting the involvement of alternative signaling mechanisms. One major pathway involves cyclooxygenase (COX) modulation. Endogenous CO enhances COX enzymatic function in hypothalamic neurons and astrocytes, leading to increased prostaglandin E_2_ (PGE_2_) synthesis. In parallel, CO interacts with mitochondrial hemoproteins, including cytochrome c oxidase, inducing a mild and reversible modulation of oxidative phosphorylation. This controlled effect promotes adaptive mitochondrial signaling, thereby supporting antioxidant and cytoprotective responses. Carbon monoxide also targets large-conductance calcium-activated potassium (BK_Ca_) channels, particularly in peripheral chemosensory structures. Under normoxic conditions, continuous CO production by HO-2 maintains BK_Ca_ channel activity and membrane repolarization. During hypoxia, reduced CO synthesis leads to channel closure, membrane depolarization, enhanced neurotransmitter release, and increased ventilatory drive. Finally, CO modulates intracellular signaling pathways, including p38 mitogen-activated protein kinase and phosphatidylinositol-3-kinase/Akt, and regulates transcription factors such as hypoxia-inducible factor-1α and peroxisome proliferator-activated receptor-γ. This has contributed to the integration of metabolic, inflammatory, and stress-adaptive responses in neural cells.

Early investigations have identified the hemoprotein soluble guanylyl cyclase (sGC) as a principal molecular target through which CO mediates its biological actions in the nervous system, leading to elevated intracellular cyclicGMP (cGMP) concentrations (Figure 7) [15]. However, subsequent studies challenged this notion, demonstrating that CO is only a weak activator of sGC. Indeed, compared with nitric oxide (NO), which can enhance cGMP synthesis by up to two orders of magnitude, CO elicits only a modest 2- to 4-fold increase [91,92,93]. Along with earlier findings on the interplay between NO and cyclooxygenase (COX) pathways, these observations suggested that the neuroendocrine effects of CO may instead involve modulation of COX activity, an enzyme abundantly expressed throughout the brain, including the hypothalamus [94,95,96,97]. Our research provided direct evidence supporting this hypothesis, revealing a close correlation between endogenous CO production and COX enzymatic activity. Specifically, hemin exposure significantly enhanced COX activity in rat hypothalamic explants and in primary cultures of hypothalamic astrocytes [98]. The increased prostaglandin E_2_ (PGE_2_) synthesis was abolished by the selective HO inhibitor Sn-mesoporphyrin-IX (Sn-MP-IX), and reversed by the CO scavenger hemoglobin, confirming that COX activation was dependent on HO-derived CO [98]. Consistently, incubation of rat hypothalamic explants in CO-saturated media also resulted in a marked elevation of PGE_2_ release [36].

Carbon monoxide also interacts with other hemoproteins, including neuroglobin and cytochromes, influencing cellular oxygen consumption and redox balance [35,99,100]. In particular, endogenous CO interacts with mitochondrial cytochrome c oxidase (Complex IV) in a reversible and finely regulated manner [101,102]. Unlike the inhibitory effect observed during poisoning, physiological CO levels cause a mild and transient modulation of mitochondrial respiration that can trigger adaptive responses. This includes a controlled increase in mitochondrial ROS production, which activates redox-sensitive transcription factors, such as Nrf2, that stimulates antioxidant and cytoprotective gene expression [103,104,105]. Jointly, these interactions contribute to the preservation of neuronal metabolic homeostasis and can support cell survival under transient hypoxic conditions.

A further molecular target of CO is the family of large-conductance calcium-activated potassium channels (BK_Ca_), along with other potassium channels such as K_ATP_ channels [33,106] (Figure 7). In particular, the BK_Ca_ channels function as key effectors in the CO-dependent modulation of ventilatory responses to hypoxia [71,107]. Under normoxic conditions, HO-2 tightly binds heme and continuously produces CO. The locally generated CO activates BK_Ca_ channels, promoting potassium efflux and maintaining membrane repolarization. When oxygen availability decreases, as during hypoxia, the affinity of HO-2 for heme is reduced, leading to diminished CO synthesis. Lower CO levels cause BK_Ca_ channel closure, which restricts potassium efflux and results in membrane depolarization. This depolarizing shift enhances the release of neurotransmitters—including acetylcholine, dopamine, and ATP—from carotid body chemoreceptor cells, leading to an increase in both the rate and depth of ventilation [90,108,109].

Lastly, endogenous CO modulates signal-transduction cascades, including the p38 MAPK and phosphatidylinositol-3-kinase/Akt pathways, and influences transcription factors, such as HIF-1α and peroxisome proliferator-activated receptor-γ [110,111] (Figure 7). Through these routes, CO regulates inflammatory gene expression, promotes anti-apoptotic signaling, and contributes to the adaptive stress response in neurons and glial cells.

## 4. Carbon Monoxide and the Stress Axis

### 4.1. Carbon Monoxide and the Hypothalamic-Pituitary Unit

The first evidence linking CO to the regulation of the HPA axis was reported in 1994. In that study, hemin administration did not modify basal CRH secretion from rat hypothalamic explants, but markedly suppressed CRH release when stimulated by K^+^-induced depolarization or IL-1β [31]. This inhibitory effect was referred to CO formation, rather than to hemin itself, since it was significantly attenuated by Zn-protoporphyrin-IX (Zn-PP-IX), a selective inhibitor of HO activity [15,31,112]. The central role of CO in stress axis regulation was subsequently reinforced by several studies. These investigations demonstrated that (i) hemin is metabolized to BV and CO within the rat hypothalamus under the same conditions that inhibit stimulated CRH release, and (ii) the increase in CO formation accounts for the suppression of K^+^-evoked AVP and oxytocin release from hypothalamic explants in vitro. This effect was specifically mediated by CO, as it was reproduced by direct exposure of hypothalamic tissue to CO-saturated medium [113,114,115]. Notably, neither BV nor BR altered basal or stimulated neuropeptide release, further supporting the specificity of the CO action [114,115]. The involvement of CO in immune-inflammatory stress regulation was later confirmed by in vivo experiments. Such studies were technically demanding due to the poor permeability of metalloporphyrins, including hemin, across the blood−brain barrier at pharmacologically relevant concentrations. To overcome this limitation, intracerebroventricular (i.c.v.) administration was employed. Under these conditions, Sn-protoporphyrin-IX (Sn-PP-IX) and Zn-deuteroporphyrin 2,4-bis glicol (Zn-DPBG), two well-established HO inhibitors, significantly potentiated the LPS-induced increase in circulating AVP while reducing its hypothalamic stores [116,117]. Conversely, the activation of HO through i.c.v. injection of hemin suppressed AVP release in LPS-treated rats, further highlighting the CO inhibitory effect on the stress axis [116,117]. A few studies have confirmed the role of the HO-CO system in regulating the HPA axis in animal models of psychological stress. However, findings are sometimes contradictory. As shown by El-Sayed et al., CO production significantly reduced both ACTH and corticosterone plasma levels in male albino rats exposed to cold restraint stress [118]. Conversely, both Turnbull et al. and Kim and Rivier reported that inhibition of HO activity, with a consequent reduction in CO levels, leads to a stimulatory effect on the acute ACTH response in rats exposed to intermittent electroshock [119,120]. These findings suggest potential differences in HPA axis regulation under various types of psychological stressors and highlight the need for further investigation in this area.

A frequent outcome of stress is an elevation in body temperature. During infection, fever arises from the increased production of PGE_2_, generated through the activation of central and peripheral COX-2 and amplified by pro-inflammatory IL-1β and IL-6 that enhance its synthesis [121,122,123,124,125]. Acting on neurons within the preoptic area of the hypothalamus, PGE_2_ elevates the thermoregulatory set point, thereby inducing fever [124,125]. This response is an innate defense mechanism aimed at limiting the spread or ensuring the elimination of invading pathogens [126,127]. Moreover, an increase in body temperature can also occur under conditions of psychological stress, with mechanisms not completely understood [121]. The inhibition of HO activity, by i.c.v. administration of Zn-DPBG, results in a significant reduction in LPS-induced fever in rats. This antipyretic effect is counteracted by the i.c.v. administration of a CO-saturated solution, thus confirming the role of the HO-CO system in the regulation of LPS-induced fever [128]. The mechanism by which CO induces fever during immune-inflammatory responses involves activation of the sGC/cGMP pathway, and seems to function independently of PG synthesis [129,130]. Regarding the pyrogenic effect under acute psychological stress, the inhibition of CO release, through the i.c.v. administration of Zn-DPBG, significantly reduces fever in rat exposed to 40 min restraint/immobilization, without any effect on the body temperature of non-stressed animals [131].

In addition to immune-inflammatory and psychological contexts, the role of CO extends to osmotic and metabolic stress. Reis and colleagues showed that 48 h water deprivation induces HO-1 expression in the rat SON and PVN, and that HO-derived CO enhances the firing rate of SON magnocellular neurons [132]. These early findings were supported by subsequent studies demonstrating that CO facilitates AVP release from mediobasal hypothalamic explants during hyperosmotic stimulation [133].

Although only indirectly linked to the stress axis, the influence of CO on gonadotropin-releasing hormone (GnRH) secretion deserves further attention. In in vitro studies, hemin dose-dependently increased basal GnRH release from both rat hypothalamic explants and GT1-7 mouse hypothalamic cells [134,135]. This effect was specifically abolished by either Zn-PP-IX or Sn-PP-IX, implicating the HO-CO signaling pathway [134,135]. The mechanism by which CO modulates GnRH release from the hypothalamus has long been debated. One possible explanation suggested the involvement of PGE_2_, a well-established regulator of hypothalamic neurosecretory activity [36,98]. The study by Errico et al. provided decisive evidence supporting this mechanism, demonstrating that exposure of GT1-7 cells to CORM-2 induced GnRH secretion in a time-dependent manner, associated with PGE_2_ release [134]. Together, these findings indicate that CO exerts complementary actions on GnRH and CRH secretion. The inverse relationship between stress responses and reproductive function is well acknowledged. For instance, IL-1β, a key mediator of inflammatory stress, enhances CRH and neurohypophyseal hormone release, while simultaneously suppressing GnRH secretion via direct hypothalamic mechanisms [136]. Within this physiological framework, CO appears to play a coherent modulatory role, reducing excessive activation of the HPA axis while supporting reproductive function.

### 4.2. Carbon Monoxide and Extra-Hypothalamic Areas

Endogenous CO has occurred as an important neuromodulator within stress-responsive neural circuits. In the hippocampus, CO is involved in the regulation of long-term potentiation and neuropathic pain [137,138,139]. The mechanisms involved include either the activation of the sGC/cGMP/PKG system or the inhibition of MAPK phosphorylation [137,138,139]. Carbon monoxide also attenuates endoplasmic reticulum (ER) stress and inhibits caspase-dependent apoptosis, as demonstrated in models of febrile seizure–related hippocampal injury [140]. Therefore, by preserving hippocampal integrity and functional output, CO further contributes to maintaining appropriate negative feedback on CRH release and preventing sustained HPA hyperactivation. In the amygdala, particularly within the basolateral nucleus, CO displays anti-apoptotic properties. In rodent models of systemic inflammation, treatment with CORMs or HO-1 inducers restored Akt signaling and reduced Bax expression in both the mouse amygdala and ventral hippocampus, indicating that CO engages these pathways to dampen neurotoxic responses [141]. The evidence that BNST is connected with the amygdala and hypothalamus, together with its role in acute and chronic stress response, suggests that CO also has a role in this brain area through the modulation of comparable intracellular targets, including MAPKs and redox-sensitive pathways [142,143,144,145]. As far as the role in the mPFC is concerned, this brain area exerts powerful top-down control over HPA axis reactivity and stress habituation; therefore, dysregulation of this brain area could alter HPA output [146]. Increased HO activity and HO-1 induction have been documented in the frontal/prefrontal cortex under physiological and pathological conditions, whereas HO-1 deficiency in the PFC has been associated with enhanced neuroinflammatory signatures in human disease [147,148,149]. From a mechanistic viewpoint, CO has been shown to restore MAPK signaling and enhance Nrf2-related neuroprotective pathways in the PFC of rodents with neuropathic pain or chronic kidney disease-induced cognitive impairment [138,150]. These data support the hypothesis that endogenous CO in the mPFC may facilitate resilience to stress by preserving synaptic integrity and suppressing maladaptive neuroinflammation, thereby maintaining effective prefrontal inhibition of HPA activation. As mentioned in Section 2.2, the NTS is a primary brainstem integrator of visceral afferents and exerts powerful control over autonomic and neuroendocrine responses to homeostatic and systemic stressors. Heme oxygenase-dependent CO formation has been demonstrated in the NTS, and can be upregulated by hypoxic stimuli [151,152,153]. Functional studies demonstrate that CO within the NTS regulates cardiovascular responses: microinjection of heme or inhibition of HO activity alter baroreflex sensitivity and cardiovascular responses, supporting the main role of CO in this nucleus [152,154]. With regard to the HPA axis, noradrenergic A2/NTS neurons provide a key excitatory input to PVN CRH neurons. Therefore, by modulating neuronal excitability, CO could modify the drive that NTS exerts on PVN and downstream HPA activation. Supporting this concept, experimental inhibition of HO amplifies sympathetic and cardiovascular responses, whereas HO activation attenuates these responses [155,156]. Decisive causal evidence, e.g., cell-type-specific manipulation of HO/CO in NTS neurons (particularly A2 noradrenergic cells) with simultaneous measurement of PVN activity and ACTH/GC secretion, remains an important gap and a priority for future investigation.

### 4.3. Carbon Monoxide and the Adrenal

Several biologically plausible mechanisms suggest that HO-generated CO may locally influence the function of cytochrome P450 (CYP) enzymes involved in adrenal cortisol biosynthesis, including mitochondrial CYP11A1 and CYP11B1 as well as microsomal CYP17A1, CYP21A2 [59]. This hypothesis is supported by significant evidence indicating that both HO-1 and HO-2 have been found in the fasciculated zone, where cortisol synthesis occurs [70]. Adrenal-borne CO may bind the heme moiety of steroidogenic CYP enzymes. This hypothesis aligns with previous findings in the testes, where HO-derived CO inhibited steroidogenesis in MA-10 Leydig tumor cells by binding to CYP11A, which is responsible for the conversion of cholesterol into pregnenolone [157]. Additionally, CO reduced StAR protein levels, thereby blocking cholesterol transport to the inner mitochondrial membrane and Leydig cell steroidogenesis [157]. Beyond its actions in the adrenal cortex, endogenous CO may also modulate adrenal medullary function. From a histological viewpoint, chromaffin cells express HO-2 at high levels, where it takes part in redox regulation [158]. Mechanistically, recent evidence suggests that CO could influence the synthesis or release of epinephrine and norepinephrine. Indeed, CO has been shown to inhibit the gene expression of two key enzymes in catecholamine synthesis—such as tyrosine hydroxylase and dopamine β-hydroxylase—in rat pheochromocytoma PC12 cells, probably through the inactivation of MAPK-mediated AP-1 protein-binding activities [159]. Collectively, these observations suggest that endogenous CO may serve as a local modulator of catecholaminergic output, potentially influencing the SAM component of the stress response along with its proposed effects on GC synthesis.

## 5. Carbon Monoxide and Peripheral Stress Endpoints

The role of CO in peripheral stress adaptation is well illustrated by the cardiovascular system. Carbon monoxide modulates vascular tone, endothelial reactivity, platelet function, and smooth muscle cell behavior, thereby influencing tissue perfusion under conditions of increased metabolic, inflammatory, or hemodynamic demand [23,24,33]. Although NO remains the prototypical gaseous mediator of vascular relaxation, CO may act as a complementary regulator, particularly when endothelial function is impaired or when HO-1 expression is induced as part of an adaptive stress response [16,23,32,33]. By targeting sGC, potassium channels, mitochondrial signaling, and inflammatory pathways, CO contributes to vascular adaptation to injury, hypoxia, and hemodynamic stress (see Section 3 and Section 4).

Beyond the vasculature, HO-1/CO signaling also participates in gastrointestinal and mucosal adaptation to stress. In the gastrointestinal tract, CO has been implicated in the regulation of motility, inhibitory neurotransmission, smooth muscle function, epithelial barrier integrity, mucosal defense, and immune-inflammatory balance [37,160,161]. These effects are relevant because the gut not only is a target of systemic stress but also a site capable of amplifying systemic inflammatory and metabolic responses. Experimental studies have shown that HO-1 and CO can protect against intestinal inflammation, at least in part by modulating innate immune responses and promoting bacterial clearance [162]. These lines of evidence, link CO signaling to mucosal homeostasis and host-microbial interactions.

In epithelial and lung injury models, low-dose CO has been shown to activate autophagy through mitochondrial ROS formation, suggesting that CO-dependent mitochondrial signaling may contribute to adaptive cellular remodeling under stressful conditions [163].

Overall, HO-1-derived CO represents an important mediator of peripheral stress responses that extend well beyond oxidative stress. By modulating vascular, gastrointestinal, immune, mitochondrial, and tissue-repair pathways, CO contributes to the preservation of systemic homeostasis under challenging conditions.

## 6. Carbon Monoxide Within the Gasotransmitter Network Regulating Neuroendocrine Stress Responses

It is important to acknowledge that CO signaling does not function in isolation within neuroendocrine stress circuits. Along with CO, both NO and hydrogen sulfide (H_2_S) are all produced in the brain and can act as autocrine/paracrine modulators of hypothalamic and neurohypophyseal function [164,165]. At the level of the HPA axis, available evidence indicates that these gases can modulate stress-related neuropeptide release and downstream endocrine output. Despite this fact, their effects are highly context-dependent and may vary according to the type and intensity of the stressor, the experimental model, and the species examined [166]. For example, NO and CO have been reported to facilitate the acute ACTH response to physico-emotional stressors through hypothalamic mechanisms, whereas H_2_S can modulate CRH release from the hypothalamus and influence stress-induced corticosterone responses in rats [120,167]. Mechanistically, crosstalk among these gasotransmitters may occur through reciprocal regulation of their synthesizing enzymes, convergence on shared signaling targets such as soluble guanylate cyclase/cGMP, ion channels, mitochondrial function, and redox-sensitive pathways, as well as through coordinated control of CRH, AVP, and oxytocin release [32,164,166]. Direct evidence for such interaction comes from hypothalamic-neurohypophyseal models showing that NO can acutely modulate CO and H_2_S production and thereby influence AVP, oxytocin, and atrial natriuretic peptide release [133]. Thus, CO-dependent effects in neuroendocrine stress responses should be interpreted as part of a dynamic gasotransmitter network, rather than as the action of a single isolated mediator.

## 7. Future Perspectives

In this review, we have summarized current evidence regarding the effects of CO on the stress axis. Although the regulatory role of this gaseous neurotransmitter in stress modulation is well established, several mechanistic aspects remain unresolved and deserve further investigation.

The net inhibitory effect of CO on the HPA axis described under immune-inflammatory challenges places it, at first glance, among mediators that may preserve inflammatory competence. This observation seems paradoxical when considering the substantial body of literature, partially summarized here, demonstrating the predominantly anti-inflammatory and neuroprotective properties of CO. Indeed, these autocrine effects are exerted within the same cell that produces CO in response to free radicals, pro-inflammatory cytokines, or xenobiotics, and are mediated through diverse intracellular targets (Figure 7). Conversely, CO-mediated inhibition of CRH and AVP release at the hypothalamic level, reduced pituitary ACTH secretion, and diminished adrenal GC output, reflect the coordinated HPA axis pattern typically observed in systemic immune-inflammatory conditions, such as infections. Because excessive GC release is immunosuppressive and may prolong or exacerbate infectious processes, CO-mediated limitation of HPA axis activation may actually represent an adaptive mechanism. Moreover, the pyrogenic effect of CO may further contribute to microbial clearance. Through these paracrine mechanisms, CO may help maintain appropriate immune competence and facilitate the timely resolution of infections.

The integrative function of CO is further supported by findings obtained under metabolic impairment, such as hyperosmotic stress induced by water deprivation. Under these conditions, CO enhances hypothalamic AVP release, which promotes renal water reabsorption and stimulates adrenal cortisol secretion [168,169]. While the action of AVP on renal water conservation is clear, the physiological rationale for the concurrent increase in cortisol is more complex. Cortisol supports essential metabolic and cardiovascular adjustments during dehydration by promoting gluconeogenesis, maintaining vascular tone, and modulating renal water handling [59]. In this context, CO coordinates osmoregulatory and HPA axis responses to optimize water conservation and energy mobilization during hyperosmotic stress.

A major unresolved issue concerns the role of CO in extra-hypothalamic regions that shape the magnitude, duration, and termination of HPA axis activation. Although most available studies have focused on hypothalamic and neurohypophyseal mechanisms, much less is known about CO signaling in corticolimbic and brainstem nodes that regulate stress integration. In particular, the BNST and NTS remain underexplored. As mentioned above, the BNST is a key interface between limbic inputs and hypothalamic stress-effector systems and may be especially relevant for sustained or anticipatory stress responses. Future studies should serve to clarify whether HO/CO signaling in this region differentially modulates GABAergic, glutamatergic, and CRH-expressing neuronal populations, and whether these effects vary according to acute versus chronic stress exposure. Similarly, the NTS represents a critical relay for visceral, autonomic, and immune-inflammatory signals reaching the PVN. Defining how CO influences catecholaminergic NTS neurons, vagal-afferent processing, and immune-to-brain communication may help clarify how peripheral inflammatory or metabolic challenges are translated into coordinated neuroendocrine and autonomic outputs.

Another important gap concerns the transcriptional regulation of HO-1 following psychosocial stress. Evidence indicates that acute restraint stress induces free-radical production and pro-inflammatory cytokine release [170,171]. These stressors have been shown to upregulate the HO-1/CO system through Nrf2- and NF-κB/AP-1-dependent pathways. While these findings are convincing, they remain incomplete, and in-depth studies are needed to clarify the specific contribution of these transcription factors to HPA axis modulation during psychosocial stress. In this regard, future work should move beyond global pharmacological approaches and adopt cell- and circuit-specific strategies.

Conditional manipulation of HO-1 and HO-2 gene expression, in defined neuronal and glial populations, viral-mediated overexpression or silencing of HO enzymes, and region-selective approaches targeting the PVN, BNST, NTS, amygdala, hippocampus, and mPFC would help distinguish the source, target, and timing of CO signaling during different stress modalities. These approaches should ideally be combined with neuroendocrine readouts, including CRH/AVP release, ACTH and GC secretion, autonomic parameters, inflammatory markers, and behavioral outcomes.

From a pharmacological and translational perspective, the HO/CO pathway represents a promising therapeutic target. As discussed in Section 3.2, HO-1 inducers, HO inhibitors, gaseous CO, and CORMs have all been used experimentally to manipulate this pathway, but each strategy presents important limitations related to specificity, dose, tissue penetration, kinetics of CO release, and possible CO-independent effects. An additional caveat concerns strategies aimed at chronically enhancing endogenous CO production through sustained HO-1 induction. Such strategies include nutraceutical approaches based on polyphenol-mediated activation of the Nrf2/HO-1 pathway, which have been proposed in several models of aging and neurodegeneration, including Alzheimer’s disease [172,173,174]. Although such interventions may promote adaptive cytoprotection, HO-1 activation is not equivalent to selective CO delivery, because heme degradation also generates BV/BR and Fe^2+^ [16,20,22]. Therefore, prolonged or poorly controlled HO-1 upregulation may become maladaptive if not accompanied by adequate iron sequestration/export, ferritin induction, and BVR activity, particularly in vulnerable brain regions affected by aging, chronic stress, or neurodegeneration [175,176]. Future studies should clarify whether counter-regulatory mechanisms, including Bach1-dependent repression of HO-1 gene and other transcriptional or epigenetic feedback loops, act as safeguards against excessive HO-1 activity and heme-catabolite imbalance [78,79,80,81]. Such research work may have translational relevance for stress-related psychiatric and metabolic disorders characterized by altered HPA axis activity, neuroinflammation, autonomic imbalance, or impaired energy homeostasis. However, any therapeutic extrapolation must remain cautious, given the narrow biological window separating adaptive CO signaling from toxic interference with oxygen transport and mitochondrial respiration.

## 8. Conclusions

In conclusion, the evidence reviewed herein highlights endogenous CO as a multifaceted neuromodulator of the HPA axis, exerting context-dependent actions that integrate neuroendocrine, immune, and metabolic responses. A more precise understanding of the spatial, cellular, and temporal dynamics of HO/CO signaling in hypothalamic and extra-hypothalamic stress circuits will be essential to define its physiological relevance and its potential translational value in stress-related neuropsychiatric and metabolic disease.

## Data Availability

No new data were created or analyzed in this study. Data sharing is not applicable to this article.
